# Supplementary material for: Histological Identification and Quantification of Eosinophils and Ascites in Leghorn Chickens Treated with High Oral Concentrations of NaCl–Pilot Study
Source: Toxics. 2022 Jul 9;10(7):381. doi: 10.3390/toxics10070381 (PMC9324580; doi:10.3390/toxics10070381)
Supplement: Supplementary file 1 [file toxics-10-00381-s001.zip › toxics-1776970-supplementary.pdf]

toxics

Supplementary Materials: Histological Identification and Quantification of Eosinophils and Ascites in Leghorn Chickens Treated with High Oral Concentrations of NaCl–Pilot Study

Victor M. Petrone-Garcia, Inkar Alejandro Castellanos-Huerta, Saeed El-Ashram, Marco A. Juárez-Estrada, Benjamin Fuente-Martínez, Danielle B. Graham, and Guillermo Tellez-Isaias

Table S1. Ingredient composition (kg) and nutrient contents of feed supplied to the experimental SPF chickens.

Ingredients	Inclusion (kg)
Yellow corn 7.1%	622.62
Soybean meal 46.5%	323
Limestone 38% Ca	18
Phosphate 21/27%	12
Vegetable oil	10
NaCl (refined salt)	4
Vitamin premix (Laying chicken GT V1, Trouw Nutrition, Zapopan, Jal., Mexico)	1.4
Mineral premix [6]	1.1
DL-Methionine 99% (MetAMINO ®, Evonik, Essen, Germany)	3.700
Liquid L-lysine 50% (ADM, Chicago, IL, USA)	3.500
L-Threonine (ThreAMINO ®, Evonik, Essen, Germany)	0.640
6-Phytase (Axtra PHY TPT 10000 ®, DuPont Industrial Biosciences, Marlborough, UK)	0.045
**Nutrients**	
Weight	1.0
Dry matter (%)	88.300
Crude protein (%)	20.000
Metabolizable energy (Mcal kg^{-1})	3.087
Choline (mg kg^{-1})	2.000
Arginine (%)	1.210
Linoleic acid (%)	1.200
Total lysine (%)	1.150
Total calcium (%)	1.050
Methionine + cystine (%)	0.830
Valine (%)	0.830
Total Threonine (%)	0.820
Isoleucine (%)	0.790
Total methionine (%)	0.510
Phosphorus available (%)	0.480
Digestible phosphorus (%)	0.440
Total tryptophan (%)	0.210
Total chlorine (%)	0.200
Total sodium (%)	0.200
